# An Analysis of Guislain's Work on Insanity
*"Lectures, Theoretical and Practical, on Mental Diseases, being a Course delivered at the Clinique of the Asylums for the Insane at Ghent." By J. Guislain, Professor in the University of Ghent. 1852. 3 volumes.


**Published:** 1853-04-01

**Authors:** 


### AN ANALYSIS OF GUISLAIN'S WORK ON INSANITY.*

### No. I.

The author informs the public that these lectures were delivered *extempore*, in the midst of a population of lunatics; and that they were collected by Dr. Vermeulen, his assistant. He has published them in all the simplicity, or rather, as he says, in all the *naïveté*, of their original form. M. Guislain admits, however, that upon several points where it was required by the actual state of science he has made additions.

In the ample analysis which we propose to make of this work, we think it desirable to retain, as far as practicable, the graphic and didactic character of the original; and with the view of better preserving the author's arrangement of his subject, we find it convenient to retain the division into lectures and parts. The first volume contains seventeen lectures, and embraces the mode of examination of patients and the diagnosis of insanity; the classification of the forms of insanity; melancholy; ecstasy; mania; alienations which may be comprised under the denomination of insanity, including suicide; delirium; dementia. Then follows the anatomical diagnosis—the morbid anatomy.

The second volume treats of the occasional and predisposing causes of mental diseases; the utility of establishing a mental pathogeny; and the prognosis of mental diseases.

In the third volume the treatment is considered at great length. This concluding volume also contains descriptions of various instruments or apparatuses subservient to treatment; and several lectures are devoted to the subject of the construction and organization of lunatic asylums.

The *first lecture* commences with a general survey of the study of mental diseases. The author relates the difficulties he encountered at his outset in the investigation of these diseases. "I have spent," he says, "ten years of my life in interrogating the living man and the dead body; ten more have been given up to meditation upon what I saw: during the latter years only have I learnt how to cure the insane." He forcibly lays before his pupils the

* "Lectures, Theoretical and Practical, on Mental Diseases, being a Course delivered at the Clinique of the Asylums for the Insane at Ghent." By J. Guislain, Professor in the University of Ghent. 1852. 3 volumes.

advantages attending an accurate acquaintance with insanity, reminding them of the numerous occasions which arise to call for the decision of the physician.

He thus refers to the position of the insane in Belgium :—"Strange things pass in this country: hitherto the insane have had nothing but vows and words, for the most part sterile enough; they are pitied, but that is all." "Even the capital has no asylum for the insane!" Years pass on, and no one hears the complaints of these unfortunates. They remain forgotten, shut up in gloomy prisons. They are the object of an infamous traffic. Between the commune, the province, and the government, nothing is done: all evade a troublesome question. "The physicians who specially cultivate the study of mental diseases are limited in Belgium to a very small number. I have thought that by forming a band of able men, by showing what may be done for the holy cause in which we are engaged, there will arise a means of hastening the advance of reform. For ten years this course ought to have been given, but an obstacle has always interposed. Must it be spoken? Your presence, the presence of young men, has been feared, in the midst of this population of afflicted patients. I have spoken of you as I ought; I have combated erroneous opinions ; I have overcome this difficulty. Thanks to the enlightened and powerful intervention of the Administrative Inspector of the University, and the efforts of the Commission des Hospices, I have succeeded. It is for you, gentlemen, so to conduct yourselves that there be no room for reproach: you must have prudence; you must not address indiscreet questions to the patients; you will wait till I invite you to examine them; you must not excite them by your discourse, or by your looks; they must not feel your presence." M. Guislain says that twenty pupils were admitted at a time; and he bears the same pleasing testimony to the result which is recorded by M. Falret and other physicians, who have pursued a similar course. "Their presence," he says, "has not given rise to the smallest inconvenience; on the contrary, their arrival was always followed by a marked good effect among the patients."

*How to proceed in the practical examination of the insane.*—The practical examination of a lunatic is altogether different from that of a patient suffering simply from a bodily disorder. The direct investigation of the organs loses immensely in value. You do not say to the lunatic, "Where are you in pain? What do you complain of? How long have you been ill?" This is what he will answer you—"I am in no pain. I am very well. What do you want with me?" Or else he does not answer at all; or his speech is out of joint.

We are but imperfectly acquainted with the condition of the diseased organs of the insane ; we are but imperfectly acquainted with the functions of the brain. The anatomical knowledge of this organ fails to guide us to the knowledge of the seat of these functions. But if we know not the seat of the intelligence, of the impulses, of the passions, we know that there are these functions; we are conscious of a personal identity and of passions. First of all, I must address myself to these manifestations, and not to the cerebral pulp. It results from this truth, that *symptoms* are of extreme importance in the analytical appreciation of mental alienation. You will more frequently interrogate these symptoms than the brain or the alterations of its tissues. You will practise the analysis of the functions of the intellect, the physiognomical expression of the passions, the value of ideas, the bearing of acts and words. You will do all this, taking for your guide facts and the living man.

These must be your finger-posts in the clinical study of mental diseases:

I. The physiognomy.
II. The action.
III. The speech.
IV. The viscera.
V. The commemorative history.

A. *The physiognomy.*—As the very foundation of the estimate of a patient, you must bring to bear the medical *coup d'œil.* I will define it: it is the art of seeing in an assemblage of phenomena a crowd of details where others see nothing but generalities, and sometimes nothing whatever. But the *oculus medicus*—do not deceive yourselves—is only a reality when it presents itself as the fruit of practice and of study. Do not believe that the most subtle, the rarest intelligence, will recognise a given disease better than the most ordinary physician, if this intelligence has not been initiated into the secrets of science and of observation, and if it knows not how to transform into scientific ideas the impressions supplied by the senses. Tact is only developed in the physician by time. He learns quickly enough to recognise the signs of certain organic alterations—that is, the science of the amphitheatre. But the purely dynamic disorders are entirely different. It takes long years to qualify him, in a prognostic point of view, to judge well of the curability or incurability of diseases. In the midst of all this there is nothing like a compass, and in this respect the cardinal points I have indicated are sure guides.

Before thinking of any plan of treatment, the physician must submit the lunatic to sustained observation; he must dwell upon numerous sources of information. In the presence of his patient he seeks, in some way, to discover the impression he himself produces upon him. He sees him much, and for a long time, by day, by night; and it is only at the end of some time that he will know him, and be able to decide upon the character, the nature, and the issue of the disease. This observation—do not lose sight of it—is, above all, important in cases of medico-legal investigation.

The assemblage of phenomena, the details of the features, the attitude of the patient, his gesture,—this, above all, must claim your attention. It is the expression of the face which will reveal to you the emotions, the passions, which govern the lunatic. Every kind of alienation has its *facies.* Every madman has his features, his peculiar gestures. These features are so many signs which direct you in the appreciation of what is going on within. This expression of the face I will call the *mask of mental alienation.* It is eminently significative; it alone can show whether a person is or is not insane. The pantomime relates to the general gesticulation, and is not less important.

It is extremely useful to know the different shades of this play of the physiognomy, in order to appreciate a predisposition; to determine the existence of insanity at the outset; to determine the transition from one form of alienation to a different form; when there is a question of liberating a patient who has recovered; in the case of medico-legal investigation of simulated insanity, for example, and on a hundred other occasions.

The mask furnishes different signs:—First, the complexion; then the condition of the hair—its greasy covering, its firmness, its direction; the signification of the lines which mark the forehead and the cheeks, the eyes—the mirror of the soul; the mouth, and the movements of the tongue; the general expression of the features, the physiognomy.

I will bring before you a series of patients. Here is one whose eyes indicate the disorder which reigns in his understanding. His fixed looks hardly change their direction; winking takes place only at long intervals. In this other patient all the lines of the face are strongly marked; there is something very strongly drawn in the eyebrows, in the furrows which mark the cheeks and forehead.

The abnormal contraction of the *muscles of the face* alters the features to such a degree, as often to render the patient not recognisable. He appears older; he is uglier than before his illness. This is the reason why we seldom meet with handsome faces in lunatic asylums. In convalescence, when the morbid tension ceases, the features are more regular, the skin acquires freshness, the eye is more calm, the wrinkles disappear.

The folds of the forehead have a speaking meaning; they announce affliction, anxiety, and moral suffering. The lines which define the eyebrows, the eyelids, the eyes, furnish the most precious indications. Astonishment, anger, jealousy, hatred, translate themselves in the eyebrows and the eyes. The aspect of the eyes alone sometimes suffices to disclose a leaning to suicide. In effect, there is in the look of that patient whom you see there, a quite peculiar expression, which, added to the bluish tint of the lips, gives to his face a something that is frightful. He is a madman who seeks to destroy himself.

Grief reveals itself in the *eyes*. The eyes alone announce this condition. Irritation, discontent, wants, are also read there, as you may witness in the maniacs around you. This epileptic bears in his astonished, vacant, stupid look, in his open eyes, the characters by which a practised eye may recognise him at a glance.

The *Features*. In certain conditions the face seems to swell, the nervous centres seem to cease to innervate the muscles. Often during the passage from one form of alienation to another, we see a relaxation spreading over all the muscles of the body. This condition is not a paralysis in the strict acceptation of the word, but it constitutes a condition bordering upon paralysis. It implies a giving way of the state of tension, a want of nervous influx of tone.

Here is a patient who exhibits a marked change in the complexion of his skin, which has become olive-brown. This sign is important when the question arises, whether a particular patient is in a fit state to be restored to his family. It often occurs to me to say: this man must stay here a little longer; his skin has not recovered the complexion of health.

Here is another patient who shows pallor, especially whiteness of the lips. This sign has its importance; it indicates concentrated passions.

The *Hair*. In serious cases, the hair undergoes a striking alteration of colour, of texture. Black hair acquires a reddish tinge, as if it had been dyed. Light hair grows pale; I have seen it sometimes look as if it had been burnt, breaking at the slightest touch, leaving the cranium bare, whilst the root remained in the bulb. Sometimes the hair becomes woolly, or silky. I have seen it sometimes quite dry at the ends, whereas the patients naturally had it very greasy.

The *movement of the tongue* deserves especial attention. Slowness of speech, a defect in accentuation, hesitation in pronunciation, disorder in the succession of the words, are so many phenomena of great value in diagnosis. They indicate very serious cases. The patient I now present to you is struck with what is called general paralysis: I wish you to observe the hesitation he manifests in pronouncing his words, and in linking his phrases. These signs are important in prognosis: they announce the extreme gravity of the disease, and the probable existence of alteration of the cerebral tissue.

B.—I will now present a series of patients, illustrating the meaning of *attitudes*, *gestures*, and *movements*.

From the examination of the face, you pass on to that of the general bearing. The *locomotive muscular system* is to the moral principle what the tongue is to gastric affections. It is, if I may so express myself, the pulse to be consulted in mental derangements, when we seek to determine the condition of the force of the *sensorium commune*. Instead of seizing the wrist, as in the examination of any other disease, the physician will direct his attention to the muscular action, especially to that of the extensors. It is by the appreciation of the locomotive acts that you will often be able to determine the degree of curability, or of incurability of the disease.

Cerebral excitement, exhaustion of the moral forces are, expressed by the action of the voluntary muscles. You will find in all lunatic asylums a certain number of maniacs who refuse to seat themselves upon the benches or chairs, but whom you will always meet *crouched down*, with the chin resting on the knees. This position announces a fatal progress of the disease, an enormous diminution from the *quantum* of curability.

The *inclination of the head forward* is almost the first indication of an incurable dementia; it is owing to relaxation of the extensor muscles of the neck.

When you have examined the patient in order to ascertain the extent of his disease, you will also examine him with reference to the *state of his strength*, and in this respect, I repeat it, it is not the cardiac pulse, but the pulse of locomotion which will guide you.

But the condition I have just described must not be confounded with other conditions which may present a certain analogy. There is in the insane a tension, an immobility which must be distinguished from the muscular relaxation of weakness and paralysis. In many insane patients who appear to be in a state of prostration, there is a muscular tension. In taking the hand, the arm of the patients, you experience a certain resistance, a certain difficulty in extending the limb. This condition is far from possessing that signification which it presents in the case of which I have just spoken; it announces an irritation of a peculiar kind of the nervous system.

Nothing is more rare among the insane than partial paralysis of the muscles of the face or of the limbs. You will seek in vain in this establishment for contortions of the mouth, partial dropping of an eyelid, deviations of the tongue. You will witness muscular prostrations, paralysis of the whole life of relation; you will also see convulsions, epileptic or epileptiform; but isolated paralysis, the paralysis of a muscular group, is what will seldom be observed.

Occasionally there exists an astonishing energy of muscular action. This is the result of mental exaltation.

What is not less worthy of attention is the ease, the suppleness, the co-ordination with which all the movements of the body are accomplished. This state often presents itself as the precursory symptom of a more violent state. It announces, during the lucid intervals, that a maniacal attack is about to explode, and during convalescence it is the forerunner of complete restoration.

In others, an *exciting principle* flows from the centres like a motor influx. It is not a muscular excitement, an exaltation of the irritability: in similar cases we should rather have the convulsive mode. But here the action is not spinal, it is cerebral, mental; it consists in impulses continually transmitted to the instruments of locomotion.

The *gesture* often betrays the passion which governs the patient. Every passion has its gesture. The erotic madman affects a languishing air. The proud madman is recognised by his bearing; his head drawn up, and the stiffness which reigns throughout his frame. The religious madman proclaims himself by a peculiar attitude of humility and of concentration.

Sometimes there is nothing but this appreciation of the gesture to aid the physician in determining the real situation of the patient. This exterior expression reflects the internal state with striking accuracy. Thus there are situations in which the insane patient refuses to answer; others in which he speaks a language you do not understand. A short time ago a boy was brought to me; he spoke a gibberish which no one here understood. The directors of the prisons considered him insane; the administration of the town believed him a vagrant. The police wished for a decision. It was necessary to decide the question by answering yes or no. I said yes, he belongs to the insane. I was guided by the exterior expression. This lad had the air of imbecility; he carried his hands in his pockets, his head on one side; he did not look at me, but almost turned me his back; one foot was turned in, the other out. He came as if out of a sleep when I spoke to him. He was an idiotic vagrant who had passed the frontier; he had come from France, and had been arrested by the authorities.

### Second Lecture.

C.—*Appreciation of the Speech.* When an insane patient is presented to you who announces in his exterior neither sorrow, nor discontent, nor imbecility, nor joy, nor fear, most often you will not fail to discover a serious disorder. Nothing is so astonishing as the *answers.* Scarcely has this patient, upon whose countenance there is nothing to betray aberration, uttered a single word, than we at once understand his disease. It is an accusation against some authority or another—against his brothers or sisters. They have, he says, cast a spell upon him; they have made him miserable.

We must learn to familiarize ourselves with the *discourse* of the insane. We must learn to seize the morbid expression inherent in their speech. When the madman says he is a lost man, that he has offended heaven, that he has failed in his duty, you must for the most part believe nothing of what he says. These are pathological phrases.

The question calls for a still more serious examination when we have to ascertain the reality of a cure, and to restore the patient to his home. His disease may appear to have left him; he speaks not a word that is irrational; he is thought to be cured. But he passes by you without saying "Good morning;" he remains quietly retired in his room; he does not come forth to meet the physician; he refuses to see an old friend, a relation. His countenance betrays a certain irritation. His disease is in a manner *condensed.* You speak to him: "He knows (says he) *their* machinations; he is not *their* friend; he has been made aware of all. He knows there are freemasons around him; that there is a God; that he is not of those who have no religion." This patient is not healed; from time to time his conversation betrays the disorder, which will break out.

The speech sometimes expresses nothing but *confused and incoherent ideas:* but in othersit announces a remarkable clearness in the ideas, although they are delirious. One would say, that the faculty of creating images had doubled or tripled in energy. We must, therefore, learn to dive into the domain of ideas, and to discover there the morbid conceptions. For this purpose, we must take for our guide the great motives which determine human actions.

We must sound in the direction of erotic ideas, of the ideas of religion, of propriety, of progress, of ambition. We must stir up motives deeply hidden; we must run through the links of the many misfortunes which afflict mankind. It is in this field that you will work, and then you will make important discoveries. I will use a metaphor to make myself understood. You must cast the plummet into the receptacle of the feelings, of the ideas, of the passions. You must morally percussate the understanding. You must know how to explore the moral pulse.

You will consult the different functions of the intellect. You will seek for information in the ideas, the reasoning power, the judgment, and the power of calculation. You will sound the memory to its very depths. You will let the imagination speak. You will address yourself to the will, to the attention. Above all, you must ascertain the condition of the *intelligence.*

But what is the value of this term? what is the function it indicates? Intelligence is taken in two acceptations: as a general term; as a special function. We consider it here under the latter point of view. The intelligence is not the reasoning power; it is not the judgment; it is an appreciating sense, a psychical sense which perceives, understands suddenly, without effort. As soon as there is effort, calculation, weighing, there is reasoning.

You will, therefore, interrogate the patient, to learn the state of his intelligence, that of all his mental faculties. You will seek to learn how far the faculty of understanding is impaired; is he conscious of his position? does he know that his mind is disordered? has he notions about the cause, the invasion, and the progress of his disease? He has intelligence if he knows how to keep his room in order; if he takes care of his clothes; if he accounts to himself for what he sees. But his intelligence may be impaired in one point, and remain intact in a large sphere of mental operations.

A patient may show a perfect intelligence for all the objects which strike his senses, he may be perfectly intelligent as to all that constitutes his relations, his external impressions; and yet he may be quite unable to understand an abstract motive, or his own situation as a lunatic.

This is often the *punctum cæcum* of the *intellectual retina.* We shall see that a man may be insane, and yet not cease to be intelligent. If the faculty of understanding is weakened, this condition re-acts upon the questioner, and generally causes him to raise his voice. Here, then, is a moral thermometer, which marks the degree of conception possessed by the patient. There is a deafness of the intelligence, as there is a deafness of the ear.

The patient who no longer recognises his brother, his sister; who knows not where he is; who no longer knows that three and three make six; who when you speak white, answers black; has lost intelligence, and at the same time memory, and the reasoning power. In this situation he may present regular features, and retain the integrity of the functions of the senses. He may see his father, and not know him; he may see him die, and feel no emotion.

The absence of *moral liberty* may be deduced from the general acts of the patient, from his extravagances, and his errors. It may also be deduced from his answers; as when he tells you he cannot conduct himself as he would wish. It may be deduced from the trials to which you will subject him. You will promise him his liberty, on condition of his ceasing to exhibit such or such an idea, or act. He will not be able: the morbid manifestations will always return, in spite of his desire to repress them.

In the interrogations to which you subject the patients, there is one point to which it is important that I should direct your attention: it is the *memory.* Frequently this faculty presents an astonishing exaltation. This condition usually coincides with a general exaltation of the ideas and of the will. It properly belongs to mania. It is enough to make the patient talk, to be assured of this condition. Let it be observed, that so long as this exaltation of the memory continues, you must recognise an active state of the mental forces; you must conclude that the understanding has not yet undergone a real loss in point of strength.

On the other hand, a great impairment of the memory indicates frequently a serious disorder. It marks a great loss of intellectual energy; and often characterizes the incurability of the disease, especially when it is the expression of a chronic state, or if it accompanies hesitation of speech, and other signs of general paralysis.

In these interrogatories, it is important not to put questions at random; you must learn to direct them in such a manner as to percussate the different moral functions. You begin by gaining the good-will of the patient, and by placing him at his ease, and driving away all suspicion from his mind. Next, you endeavour to group questions around certain words, out of which you make questions interesting to the patients. These words are:

Why? How? Since when? Where? How many? The *why*, serve to measure the degree of intelligence of the patient. Why are you here? Why did you leave your house? Why don't you go home? The *how* reveals more particularly the faculty of reasoning, of the judgment. How do you do this? How do you do that? *Since when*, *where?* are addressed to the memory. *How many?* refers to the power of calculation. How much do you earn a day? and so forth.

To appreciate the value of the method I now indicate, one must have experienced how embarrassing is the position of the physician in the presence of a patient to whom he knows not what to say.

As a general rule, if a patient refuses to answer, cease to question him.

For the purpose of ascertaining how far the morbid condition has impaired the will, *that power of commanding the muscles, and of taking a resolution*, you may say to the patients: Look at me. Get up. Sit down. Take off your hat, and so on. You must press him to make his bed; to mend his clothes. You must observe which patients come to the dining-rooms at meal-times, and who do not. The laggards are men profoundly afflicted, indocile, or weakened by illness. They are characterized by a want of initiation; an inaptitude to work; and the impossibility of taking any resolution.

If the patient obeys, he manifests intelligence, and a certain freedom of will. If he gets up at the proper hour in the morning and dresses himself he has a certain amount of spontaneity.

The exaltation of the impulsive forces of the brain, of the *will*, is remarkable in many forms of mental alienation. It is the signal of the return of morbid attacks. Patients who for several months had kept themselves aloof and quiet, suddenly present themselves, pretending they must go out on business; that they must look to the purchase of a horse, an estate; that they must see their wives, their attorneys; they get up early in the morning, and are met everywhere. These patients manifest an exaltation of the will.

Do you wish to know if the patient is *attentive?* Do not lose sight of his eyes when you speak to him. If he listens to you, his visual axes meet yours; if he answers without hesitation, he is attentive. The want of attention and of will does not generally announce an exhaustion of these faculties; frequently, the incapacity is connected with functional disturbance; sometimes with a very active condition. To be enabled to say that certain phenomena are the expression of exhaustion, we must have also signs drawn from the observation of the features, and the pantomime of the patients.

D.—*The state of the Viscera.* After the foregoing examination, we have to consider the various degrees of activity, of retardation, of volume, and rhythm, which the pulse may present.

In the insane, the pulse affords no certain indications; its anomalies are but little varied, and hardly furnish information of much importance in the treatment. The pulse, however, has a certain practical value. In many cases, the patient may present all the symptoms of a state announcing a bodily disease. The exploration of the pulse alone warns the physician of the return of an attack, or informs him of the approach of complete convalescence. There is a pulse peculiar to the insane, which may be of incontestable value.

You next pass in review the viscera; you interrogate the stomach, the intestines, the liver, the kidneys, the lungs, the heart, the spinal cord, the organs of the senses; and you must learn how the patient sleeps.

E.—*The history. Facts in commemoration.*

The commemorative facts constitute one of the most important elements in the examination of the insane. They comprise the leading points of the patient's life, his education, vocation, the length of his illness, and the relapses he may have experienced. They comprise all that may elucidate the causes of the illness.

*Letters written by the insane.* The letters written by a patient supply an excellent means of learning his inmost thoughts. Even when no act or spoken word announces a morbid state, his written words often betray it. His letters frequently contain a series of expressions accurately designating the kind of disease which afflicts the patient. They are often incoherent, and full of wants. They are generally addressed to the magistracy, to the ministers, to the king. They frequently run upon the most extravagant projects. The very paper employed, the address, furnish useful indications. Thus, letters are written with a very bad pen, and full of blots; they are scraps of paper; the margins of a journal, folded with the greatest negligence; the letter is sealed with bread-crumb; it is addressed to the king, or some distinguished personage. This behaviour reveals the absence of the sentiment of propriety, a want of perspicacity; it indicates, in the majority of cases, an advanced stage of disease. Well! if you speak to the authors of these letters, upon the subject of their morbid ideas, you will see how they will break out.

*(To be continued).*

